# Supplementary material for: Spectraplakin is required for dendritic microtubule organization via a tip-based mechanism in Drosophila
Source: eLife. 2026 Nov 5;14:RP108055. doi: 10.7554/eLife.108055 (PMC13739272; doi:10.7554/eLife.108055)

Figure 6 - Figure Supplement 1 - Source data 1 uncropped

Western blots

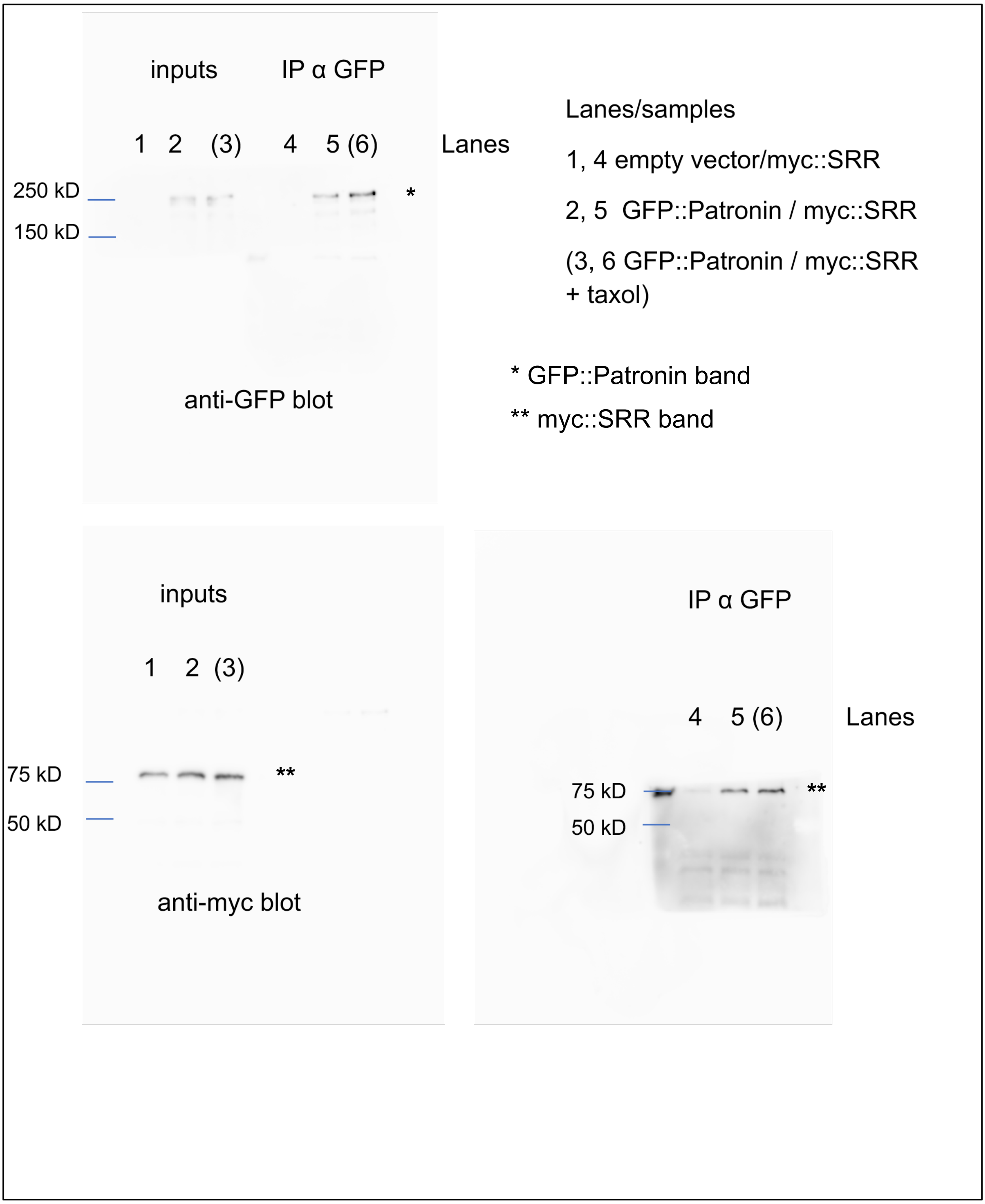

Supplement: Supplementary file 3 [file elife-108055-fig6-figsupp1-data2.zip › Figure 6 figure Supplement 1 - Source data 2 - uncropped blots with labels/Fig 6 Supplement Source data Labeled uncropped blots GFP-Patronin myc-SRR IP.pdf]
