# Supplementary material for: Spectraplakin is required for dendritic microtubule organization via a tip-based mechanism in Drosophila
Source: eLife. 2026 Nov 5;14:RP108055. doi: 10.7554/eLife.108055 (PMC13739272; doi:10.7554/eLife.108055)
Supplement: Supplementary file 5 [file elife-108055-fig7-data2.zip › Figure 7 Source data 2 - uncropped blots with labels/Fig 7 Source data Labeled uncropped blots Msps-FLAG - Rab11-GFP IP.pdf]

Figure 7 - Source data 1

uncropped Western blots

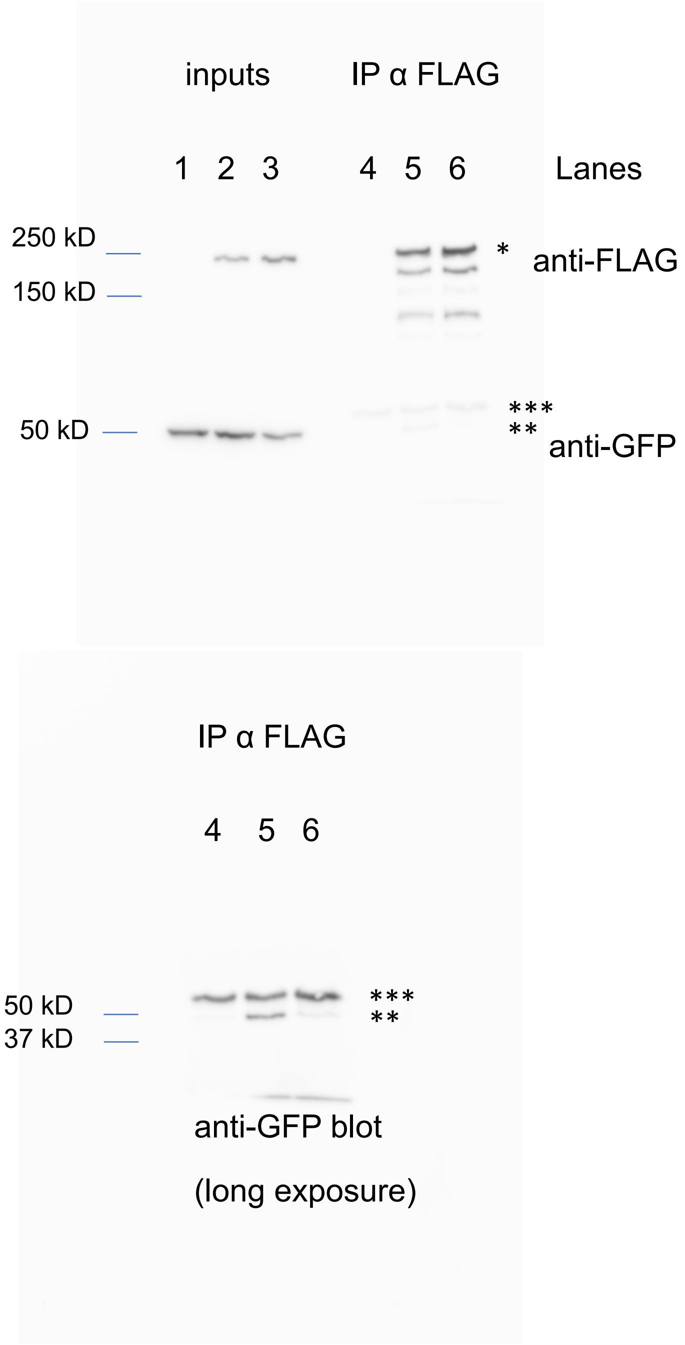

Lanes/samples

1, 4 empty vector / Rab11::GFP

2, 5 Msps-FLAG / Rab11::GFP

3, 6 Msps-FLAG / Rab11::GFP S25N

* Msps-FLAG band

** Rab11::GFP band

*** mouse IgG heavy chain

Positions of Mw markers indicated on left side
