# Supplementary material for: Spectraplakin is required for dendritic microtubule organization via a tip-based mechanism in Drosophila
Source: eLife. 2026 Nov 5;14:RP108055. doi: 10.7554/eLife.108055 (PMC13739272; doi:10.7554/eLife.108055)
Supplement: Peer review history

# Editor report: eLife Assessment

**DOI:** 10.7554/eLife.108055.3.sa0

**Authors:** Kassandra M Ori-McKenney

**Affiliations:** University of California, Davis United States

**Keywords:** Incomplete, Valuable

Davies et al. present a **valuable** study proposing that the Drosophila homologue of MACF1, Shot, can act as a molecular linker between microtubules and actin during dendrite pruning, suggesting an intriguing role in non-centrosomal microtubule organization. The study provides robust evidence that Shot helps control microtubule organization and dendrite pruning, but the evidence that Shot coordinates these processes through its interaction with actin remains **incomplete**, particularly because the actin-disrupting experiment does not affect the distal dendrites where the authors propose Shot acts. Overall, the study contains several interesting findings, but additional experimental support is needed to establish how Shot, actin, and microtubules work together to control dendrite pruning.

# Referee report: Reviewer #1 (Public review)

**DOI:** 10.7554/eLife.108055.3.sa1

The neuronal microtubule cytoskeleton is essential for long-range transport in axons and dendrites. Axon-specific plus-end-out microtubule organization versus dendrite-specific plus-end-in organization (in invertebrates) enables selective transport into each neurite, establishing neuronal polarity. Dendritic microtubule organization is also thought to be important for dendrite pruning in Drosophila during metamorphosis. However, the precise mechanisms that organize microtubules in neurons remain incompletely understood.

In this manuscript, the authors show that the spectraplakin Shot is important for developmental dendrite pruning. Shot mutants display dendritic microtubule polarity defects, which-based on the authors' rescue experiments and prior work-likely underlie the pruning defects. Since Shot is a known actin-microtubule crosslinker, the authors also examine the role of actin and find that it, too, is required for dendrite pruning.

Notably, Shot localizes transiently to dendrite tips during the 1st instar larval stage, a time when microtubule defects are already detectable. Given that Shot interacts with both plus- and minus-end microtubule-binding proteins, and that overexpression of Shot's microtubule-binding domain alone is sufficient to rescue the mutant phenotype, the authors propose that Shot's principal function is to stabilize microtubules at dendrite tips during the 1st instar stage (and possibly along the entire dendritic shaft at later stages).

In parallel, the authors investigate microtubule nucleation and find that, specifically at the 1st instar stage, microtubules can nucleate from RAB11-positive vesicles carrying γ-tubulin. Interestingly, this nucleation occurs only toward the cell body, suggesting additional layers of control. The authors do not observe nucleation from the dendrite tip, as previously reported in *C. elegans*. While this discrepancy could be technical, it more likely reflects a genuine mechanistic difference between the two systems.

Strengths:

Overall, this work was technically well performed, using advanced genetics and imaging. The authors convincingly show that Shot plays an important role in microtubule stabilization, and that its interaction with actin is likely important for localizing the protein.

The transient localization of Shot to dendrite tips and microtubule nucleation from RAB11 endosomes during the 1st instar stage is intriguing. This suggests that specific mechanisms may act during early neuronal development to establish microtubule organization, while different mechanisms maintain this organization later in development.

Weaknesses:

The link between nucleation (rab11/gamma-tubulin) and stabilization (Shot/Actin/EB/Patronin) at this stage is unclear. The authors are correct to discuss that we may be missing early events, e.g. maybe we miss early microtubule nucleation from the tip. Or alternatively could MTs frow with their minus-ends into the dendrite (see Feng et al)?

Distal enrichment of shot at 1st instar is a critical piece of data (it appears in the title). However, this was seen upon overexpression of GFP::Shot. However, does this correctly reflect the endogenous situation? Also, because its binding partner, EB1, can only be seen enriched to dendrite tips upon shot overexpression but not in controls.

# Referee report: Reviewer #2 (Public review)

**DOI:** 10.7554/eLife.108055.3.sa2

Summary:

The authors' data reveal that the spectraplakin Shot, which can interact with both actin and microtubules, is essential for the proper pruning of dendrites in a Drosophila model. A molecular basis for the coordination of these two cytoskeletons during neuronal developmental has been elusive. The authors' data support a role for Shot in regulating microtubule polarity in young neurons, and the authors link this early function to a later role in dendrite pruning. The story has many interesting components, but lacks experimental depth to solidly (and sufficiently) support the authors' claims.

Strengths:

(1) A strength of the manuscript is the authors' data supporting the idea that Shot is needed for proper dendritic microtubule polarity in young neurons and that Shot regulates dendrite pruning.

(2) Another strength of the manuscript is the data in support of Rab11 functioning as a MTOC in young larvae but not older larvae; this is an important finding that may resolve some debates in the literature. The finding that Rab11 and Msps coimmunoprecipitate is nice evidence in support of the idea that Rab11(+) endosomes serve as MTOCs.

Weaknesses:

(1) A major concern is that many of the authors' main conclusions are weakly supported. The idea that Shot exerts an effect on microtubules via an interaction with actin relies predominantly on the findings that deleting Shot's actin-interacting domains (CH domains) does not rescue the dendritic microtubule polarity defect and also alters the dendrite-tip enrichment of Shot (the change in dendrite-tip enrichment, however, appears fairly modest as Shot appears to still be localized quite well near the tip of the dendrite, if not at the actual tip). The over-expression of Mical, which was intended the disrupt actin, does not affect actin in distal dendrites in 1st instar neurons; 1st instar distal dendrite tips is where (and when) the authors propose Shot functions. It does not seem possible to draw conclusions about the actin cytoskeleton from the overexpression of Mical since this manipulation only (mildly) affects actin in the proximal dendrites of 1st instar neurons.

Given the reliance on the Shot transgenes, it is also important to test, or at least comment on, whether the Shot transgenes are expressed at equivalent levels (the transgenes appear to be integrated at different positions in the genome). A difference in transgene expression should be ruled out as a potential contributing factor.

(2) The authors propose that Shot acts by (locally) stabilizing microtubules. This conclusion is not supported by the data. For example, the fluorescently tagged tubulin images show signal that is quite diffuse, and the images are not convincing (fluorescent tubulin reports both the free tubulin pool as well as microtubules). A high-resolution image in the supplementary materials is also not convincing. These images are of primary dendrites, but the authors' model proposes a local role for Shot in dendrite tips.

(3) A general weakness is the use of data derived from different parts of the dendritic arbor, and at varying developmental stages, to generate a model that proposes a spatially and temporally specific role for Shot.

# Author comment: Author response

**DOI:** 10.7554/eLife.108055.3.sa3

**Authors:** Matthew Davies, Neeraja Sanal, Neele Wolterhoff, Ulrike Gigengack, Yitao Shen, Samuel M Frommeyer, Ines Hahn, Sebastian Rumpf

**Affiliations:** University of Münster Münster Germany; University of Münster Münster Germany; University of Münster Münster Germany; University of Münster Münster Germany; Pennsylvania State University University Park United States; University of Münster Münster Germany; University of York York United Kingdom; University of Münster Münster Germany

The following is the authors’ response to the original reviews.

**Public Reviews:**

**Reviewer #1 (Public review):**

Summary:

The Neuronal microtubule cytoskeleton is essential for long-range transport in axons and dendrites. The axon-specific plus-end out microtubule organization vs the dendritic-specific plus-end in organization allows for selective transport into each neurite, setting up neuronal polarity. In addition, the dendritic microtubule organization is thought to be important for dendritic pruning in Drosophila during metamorphosis. However, the precise mechanisms that organize microtubules in neurons are still incompletely understood.

In the current manuscript, the authors describe the spectraplakin protein Shot as important in developmental dendritic pruning. They find that Shot has dendritic microtubule polarity defects, which, based on their rescues and previous work, is likely the reason for the pruning defect.

Since Shot is a known actin-microtubule crosslinker, they also investigate the putative role of actin and find that actin is also important for dendritic pruning. Finally, they find that several factors that have been shown to function as a dendritic MTOC in *C. elegans* also show a defect in Drosophila upon depletion.

Strengths:

Overall, this work was technically well-performed, using advanced genetics and imaging. The author reports some interesting findings identifying new players for dendritic microtubule organization and pruning.

We thank reviewer 1 for this assessment.

Weaknesses:

(1) The evidence for Shot interacting with actin for its functioning is contradictory. The Shot lacking the actin interaction domain did not rescue the mutant; however, it also has a strong toxic effect upon overexpression in wildtype (Figure S3), so a potential rescue may be masked. Moreover, the C-terminus-only construct, which carries the GAS2-like domain, was sufficient to rescue the pruning. This actually suggests that MT bundling/stabilization is the main function of Shot (and no actin binding is needed).

Thank you for this comment. We agree that the rescue and overexpression experiments with UAS-Shot ΔCH1 (lacking only the first actin-binding domain) and UAS-Shot CTerm (containing only the MT binding domain) allow for other interpretations than ours that both features are necessary for Shot function in dendrites. To address this issue, we tested two more Shot domain deletion mutants, Shot ΔABD (lacking both actin-binding domains) and Shot ΔCTail, which lacks the MT binding domain (Figure 3). Neither of the latter two constructs can rescue the pruning and MT orientation phenotypes of shot mutants, confirming that actin binding is required for these functions in the context of full-length Shot. Importantly, in contrast to the previously used Shot ΔCH1, neither Shot ΔABD nor Shot ΔCTail inhibit pruning upon overexpression, ruling out other interpretations of overexpression toxicity (Fig. S2). The observation that Shot CTerm (containing only the MT binding domain) is sufficient to rescue indicates that actin binding is only required for Shot function in the context of full-length Shot. Interestingly, this is reminiscent of a proposed autoregulation mechanism where actin binding disinhibits Shot's MT binding domain (Applewhite 2013).

Figure 3, Figure S2 (Shot domain analysis): We added two new constructs (Shot ΔABD, Shot ΔCtail) and a Shot BAC as additional control in Figure 3.

(2) On the other hand, actin depolymerization leads to some microtubule defects and subtle changes in shot localization in young neurons (not old ones). More importantly, it did not enhance the microtubule or pruning defects of the Shot domain, suggesting these act in the same pathway. Interesting to note is that Mical expression led to microtubule defects but not to pruning defects. This argues that MT organization effects alone are not enough to cause pruning defects. This may be good to discuss.

Thank you for this comment. We think that microtubule orientation defects are tightly linked to dendrite pruning defects, as all known conditions causing orientation defects either cause pruning defects, or they act as genetic enhancers of pruning mutants. We now clarify this in the introduction citing the example of EB1 RNAi (causes orientation defects (Matties et al., 2010), enhances pruning mutants (Herzmann et al., 2018)). This is also the case for Mical overexpression, which causes orientation defects and enhances the pruning defects caused by Shot RNAi. To make this point clearer, we now also show representative images of pruning defects caused by either Shot RNAi alone or Shot RNAi combined with Mical overexpression.

Introduction - we explain better the genetic relationship between microtubule orientation and pruning. Figure 4 (phenotypic enhancement of shot knockdown by Mical overexpression): added representative images.

(3) For the actin depolymerization, the authors used overexpression of the actin-oxidizing Mical protein. However, Mical may have another target. It would be good to validate key findings with better characterized actin targeting tools.

Thank you for this comment. To our knowledge, there are no good tools to globally manipulate actin dynamics in vivo in a similar manner to, e. g., latrunculin in cultured cells. According to the literature, Micals are highly specific for actin. To enhance our analysis, we assessed the effect of Mical overexpression on actin distribution in c4da neurons (as assessed by LifeAct-GFP) and found significant effects at the first instar.

Added new analysis of effect of Mical overexpression on Lifeact::GFP distribution in c4da neurons (new Fig. S3), added references for Mical targets.

(4) In analogy to *C. elegans*, where RAB-11 functions as a ncMTOC to set up microtubules in dendrites, the authors investigated the role of these in Drosophila. Interestingly, they find that rab-11 also colocalizes to gamma tubulin and its depletion leads to some microtubule defects. Furthermore, they find a genetic interaction between these components and Shot; however, this does not prove that these components act together (if at all, it would be the opposite). This should be made more clear. What would be needed to connect these is to address RAB-11 localization + gamma-tubulin upon shot depletion.

All components studied in this manuscript lead to a partial reversal of microtubules in the dendrite. However, it is not clear from how the data is represented if the microtubule defect is subtle in all animals or whether it is partially penetrant stronger effect (a few animals/neurons have a strong phenotype). This is relevant as this may suggest that other mechanisms are also required for this organization, and it would make it markedly different from *C. elegans*. This should be discussed and potentially represented differently.

Thank you for this comment. We agree that the genetic interaction with pruning as readout does not prove that Rab11 and Shot act in the exact same pathway during establishment of microtubule organization. To address this question, we live-imaged Rab11::GFP vesicles in control and Shot knockdown neurons and found no significant difference in motile behavior (new Figure 7). As Rab11::GFP also is not visibly enriched at dendrite tips at the first instar stage, we cannot confidently state that a Rab11 tip MTOC exists in Drosophila neurons. Still, we only see EB1 comets originating from Rab11 puncta at the first instar, but not the third. We therefore rephrased and toned down our interpretation and state now that Rab11 may be a component of a developmentally transient MTOC but likely acts independently of Shot.

Unfortunately, we do not see many comets per neuron with our fluorescently tagged EB1 constructs, indicating that only a fraction of them are labeled. This makes it very difficult to judge severity between single neurons. The impression is that usually only some comets per neuron have reversed orientation, which would be consistent with several independent mechanisms for orientation.

**Reviewer #2 (Public review):**

Summary:

In their manuscript, the authors reveal that the spectraplakin Shot, which can bind both microtubules and actin, is essential for the proper pruning of dendrites in a developing Drosophila model. A molecular basis for the coordination of these two cytoskeletons during neuronal development has been elusive, and the authors' data point to the role of Shot in regulating microtubule polarity and growth through one of its actin-binding domains. The authors also propose an intriguing new activity for a spectraplakin: functioning as part of a microtubule-organizing center (MTOC).

Strengths:

(1) A strength of the manuscript is the authors' data supporting the idea that Shot regulates dendrite pruning via its actin-binding CH1 domain and that this domain is also implicated in Shot's ability to regulate microtubule polarity and growth (although see comments below); these data are consistent with the authors' model that Shot acts through both the actin and microtubule cytoskeletons to regulate neuronal development.

(2) Another strength of the manuscript is the data in support of Rab11 functioning as an MTOC in young larvae but not older larvae; this is an important finding that may resolve some debates in the literature. The finding that Rab11 and Msps coimmunoprecipitate is nice evidence in support of the idea that Rab11(+) endosomes serve as MTOCs.

Thank you for these comments.

Weaknesses:

(1) A significant, major concern is that most of the authors' main conclusions are not (well) supported, in particular, the model that Shot functions as part of an MTOC. The story has many interesting components, but lacks the experimental depth to support the authors' claims.

Thank you for this comment. We agree that we cannot conclusively state that Shot is part of a classical MTOC where microtubules are nucleated and therefore tone down our conclusions regarding this.

(2) One of the authors' central claims is that Shot functions as part of a non-centrosomal MTOC, presumably a MTOC anchored on Rab11(+) endosomes. For example, in the Introduction, last paragraph, the authors summarize their model: "Shot localizes to dendrite tips in an actin-dependent manner where it recruits factors cooperating with an early-acting, Rab11-dependent MTOC." This statement is not supported. The authors do not show any data that Shot localizes with Rab11 or that Rab11 localization or its MTOC activity is affected by the loss of Shot (or otherwise manipulating Shot). A genetic interaction between Shot and Rab11 is not sufficient to support this claim, which relies on the proteins functioning together at a certain place and time. On a related note, the claim that Shot localization to dendrite tips is actin-dependent is not well supported: the authors show that the CH1 domain is needed to enrich Shot at dendrite tips, but they do not directly manipulate actin (it would be helpful if the authors showed the overexpression of Mical disrupted actin, as they predict).

Thank you for these comments. In response, we tested whether Shot knockdown affects Rab11 vesicle motility in first instar dendrites and find that this is likely not the case (new Fig. 7 K, L). We therefore remove the proposal that Shot directly regulates Rab11. Regarding the actin dependence of Shot tip localization, we based this conclusion on the observations that Shot ΔCH1 does not localize to tips, and that Mical overexpression significantly broadens the Shot tip signal (Fig. 5 E, F, J - previously Fig. 5 C, D, G). To further deepen this conclusion, we now add a localization analysis of a Shot mutant lacking both actin-binding CH domains (Shot ΔABD) and find that this also abrogates tip enrichment.

(3) The authors show an image that Shot colocalizes with the EB1-mScarlet3 comet initiation sites and use this representative image to generate a model that Shot functions as part of an MTOC. However, this conclusion needs additional support: the authors should quantify the frequency of EB1 comets that originate from Shot-GFP aggregates, report the orientation of EB1 comets that originate from Shot-GFP aggregates (e.g., do the Shot-GFP aggregates correlate with anterogradely or retrogradely moving EB1 comets), and characterize the developmental timing of these events. The genetic interaction tests revealing ability of shot dsRNA to enhance the loss of microtubule-interacting proteins (Msps, Patronin, EB1) and Rab11 are consistent with the idea that Shot regulates microtubules, but it does not provide any spatial information on where Shot is interacting with these proteins, which is critical to the model that Shot is acting as part of a dendritic MTOC.

Thank you for these comments. Due in part to low overall EB1 comet density in c4da neurons, we could rarely see comets originating from tip-localized Shot::GFP at the first instar stage - one example is shown in new Fig. S5C (previously Fig. 7D). At later stages, Shot is relatively evenly localized along the dendritic plasma membrane. As we cannot be certain about the relatively vague MTOC conclusions, we decided to rephrase our conclusions and state - based on genetic and functional data - that Shot's ability to stabilize microtubules is required, likely at dendritic tips.

(4) It is unclear whether the authors are proposing that dendrite pruning defects are due to an early function of Shot in regulating microtubule polarity in young neurons (during 1st instar larval stages) or whether Shot is acting in another way to affect dendrite pruning. It would be helpful for the authors to present and discuss a specific model regarding Shot's regulation of dendrite pruning in the Discussion.

Thank you for these comments. It is indeed our hypothesis that Shot's early role in setting up microtubule organization is also crucial for pruning, this is the most parsimonious explanation as pruning and orientation defects always go hand in hand. We added the following sentence in the Discussion: "As we do not have evidence for Shot regulation at the onset of the pupal stage (Fig. S4), the function of Shot crucial for pruning is most arguably its early role in setting up uniform microtubule orientation."

(5) The authors argue that a change in microtubule polarity contributes to dendrite pruning defects. For example, in the Introduction, last paragraph, the authors state: "Loss of Shot causes pruning defects caused by mixed orientation of dendritic microtubules." The authors show a correlative relationship, not a causal one. In Figure 4, C and E, the authors show that overexpression of Mical disrupts microtubule polarity but not dendrite pruning, raising the question of whether disrupting microtubule polarity is sufficient to cause dendrite pruning defects. The lack of an association between a disruption in microtubule polarity and dendrite pruning in neurons overexpressing Mical is an important finding.

Thank you for this comment. Microtubule orientation defects are tightly linked to dendrite pruning defects, as all known conditions causing orientation defects either cause pruning defects, or they act as genetic enhancers of pruning mutants. We now clarify this in the introduction citing the example of EB1 RNAi (which causes orientation defects (Matties et al., 2010), and does not cause pruning defects by itself, but enhances the effects of other pruning mutants (Herzmann et al., 2018)). This is also the case for Mical overexpression, which causes orientation defects and enhances the pruning defects caused by Shot RNAi. To make this point clearer, we now also show representative images of pruning defects caused by either Shot RNAi alone or Shot RNAi combined with Mical overexpression.

Introduction - we explain better the genetic relationship between microtubule orientation and pruning. Figure 4 (phenotypic enhancement of shot knockdown by Mical overexpression): added representative images

(6) The authors show that a truncated Shot construct with the microtubule-binding domain, but no actin-binding domain (Shot-C-term), can rescue dendrite pruning defects and Khc-lacZ localization, whereas the longer Shot construct that lacks just one actin-binding domain ("delta-CH1") cannot. Have the authors confirmed that both proteins are expressed at equivalent levels? Based on these results and their finding that over-expression of Shot-delta-CH1 disrupts dendrite pruning, it seems possible that Shot-delta-CH1 may function as a dominant-negative rather than a loss-of-function. Regardless, the authors should develop a model that takes into account their findings that Shot, without any actin-binding domains and only a microtubule-binding domain, shows robust rescue.

Thank you for this constructive criticism. We agree that the rescue and overexpression experiments with UAS-Shot ΔCH1 (lacking only the first actin-binding domain) and UAS-Shot CTerm (containing only the MT binding domain) allow for other interpretations than ours that both features are necessary for Shot function in dendrites. To address this issue, we tested two more Shot domain deletion mutants, Shot ΔABD, lacking both actin-binding domains, and Shot ΔCTail, which lacks the MT binding domain (Figure 3). Neither of these two constructs can rescue the pruning and MT orientation phenotypes of shot mutants, confirming that both actin and microtubule binding are required for these functions in the context of full-length Shot. Importantly, in contrast to the previously tested Shot ΔCH1, neither Shot ΔABD nor Shot ΔCTail inhibit pruning upon overexpression, ruling out other interpretations of overexpression toxicity. The observation that Shot CTerm is sufficient to rescue indicates that actin binding is only required for Shot function in the context of full-length Shot. Interestingly, this is reminiscent of a proposed autoregulation mechanism where actin binding disinhibits Shot's MT binding domain (Applewhite 2013).

Figure 3, Figure S2 (Shot domain analysis): We added two new constructs (Shot ΔABD, Shot ΔCTail) and a Shot BAC as additional control in Figure 3.

(7) The authors state that: "The fact that Shot variants lacking the CH1 domain cannot rescue the pruning defects of shot[3] mutants suggested that dendrite tip localization of Shot was important for its function." (pages 10-11). This statement is not accurate: the Shot C-term construct, which lacks the CH1 domain (as well as other domains), is able to rescue dendrite pruning defects.

Thank you for this comment. Shot ΔCH1 indeed does show a partial rescue of the pruning defects (but not of the polarity defects). To clarify whether actin binding is required for Shot function in dendrites, we tested the additional Shot construct Shot ΔABD and found that this construct could not rescue the defects at all (Fig. 3).

Fig. 3, added Shot ΔABD.

(8) The authors state that: "In further support of non-functionality, overexpression of Shot[deltaCH1] caused strong pruning defects (Fig. S3)." (page 8). Presumably, these results indicate that Shot-delta-CH1 is functioning as a dominant-negative since a loss-of-function protein would have no effect. The authors should revise how they interpret these results. This comment is related to another comment about the ability of Shot constructs to rescue the shot [3] mutant.

Thank you for this comment. We agree that the rescue and overexpression experiments with UAS-Shot ΔCH1 (lacking only the first actin-binding domain) allow for other interpretations than ours - that actin binding is necessary for Shot function in dendrites. To address this issue, we tested another Shot domain deletion mutant, Shot ΔABD (lacking both actin-binding domains) (Figure 3). This construct did not rescue the pruning and MT orientation phenotypes of shot mutants, and also does not cause pruning defects upon overexpression, confirming that actin binding is required in the context of full-length Shot. As our domain analysis is reminiscent of a proposed autoregulation mechanism where actin binding disinhibits Shot's MT binding domain (Applewhite 2013), it is interesting to speculate that the overexpression toxicity of Shot ΔCH1 may stem from a residual ability of the second CH domain to relieve autoinhibition.

Fig. 3, Fig. S2, added Shot ΔABD and Shot ΔCTail to domain analyses.

**Recommendations for the authors:**

**Reviewer #1 (Recommendations for the authors):**

(1) Not enough info was given about how the statistics were performed in e.g., Figures 5E, 5H and 7C. Many data are highly significant, but this is not always directly clear to me from the images, e.g., 7C 6-10um. Or comparing 7I to 7L, how come the significance is so much different

Thank you for this comment. For quantification of the localization data, we pooled the data points from individual intensity profiles for each dendrite segment and compared them statistically. Since this generates a relatively high number of data points, samples are quite likely to be significantly different. Acknowledging this, we now only compare the most distal segments (0-2 μm, 2-4 μm). We describe our approach now in more detail in the methods section.

Better description of fluorescence quantifications in Figs. 5, 7 in Methods section.

(2) Abstract "while plus-en out MT just grow from the soma" - I think this is probably only partially correct. There are various players important for organizing/nucleating these microtubules (e.g., Augmin for local MT nucleation).

Thank you for this comment. This was oversimplified and we rephrased the abstract.

(3) "We and others have previously shown that c4da neuron dendrite pruning depends on local microtubule disassembly in proximal dendrites (Herzmann et al, 2017)." Nice to add other references.

Thank you for this comment. We added additional references.

(4) Figure 2 EB imaging. Where was this performed along the dendrite? Close to the CB, this may reflect CB MT growth entering the dendrite.

Thank you for this comment. We usually perform EB1 imaging in primary or secondary dendrites -- now mentioned in the text (p. 7). The idea that anterograde comets in this region could reflect microtubules entering from the soma is interesting, especially as the fast anterograde comets upon combined shot knockdown and Mical overexpression behave so differently. We take up this idea in the Discussion - thanks again!

(5) Figure 3H-L I cannot see the magenta in the overlays. Separate channels seem to be needed. And where is the axon? Moreover, in the text it is stated that the I 3H fusion protein is exclusively in the axon. And control is not in 3M.

Thank you for this comment. Our lacZ antibodies are relatively weak, so the axons are not always visible. We therefore changed the text to only mention the soma now. We also now include all shown genotypes in the quantification.

(6) Figure 4A I'm not sure what is seen in the image. Is this a single dendrite? And if so, why is Shot only seen on one side?

Thank you for this comment. In this panel (now moved to Fig. 5A) we had to take a fairly thin projection of a confocal stack because of strong surrounding tissue signal. Because of this, the Shot::GFP signal on one edge of the dendrite is not as well visible. We try to improve this by indicating the edges of the dendrite in the panel.

Fig. 5A - D, indicated dendrite outline in confocal/SIM images.

(7) Figure S2C description is not the same as in the graph (neurons / dendrites).

Thank you for pointing this out. We corrected the legend (now Fig. 2C).

(8) Figure S4 - is this a single dendrite or a bundle?

The images (now Fig. S5) correspond to the microtubule signal from single primary dendrites, i. e., mostly microtubule bundles. We clarify now in the legend.

Fig. S5, specified nature of samples in legend.

**Reviewer #2 (Recommendations for the authors):**

(1) In the Discussion, page 13, the authors state that: "On the one hand, we provide evidence that Shot anchors microtubules via actin in mature neurons." This is not supported by the authors' evidence; indeed, the authors themselves state on page 10 that "... the CH1 domain is unlikely to be required for Shot cortical localization in dendritic shafts." If the authors mean to say that the cortical localization of Shot is restricted to dendrite tips, then superresolution microscopy should be done on dendrite tips.

Thank you for pointing this out. As the CH1 domain is clearly not required for Shot third instar localization, we agree with this notion and rephrase our interpretation accordingly: "Our data also do not rule out that Shot could anchor dendritic microtubules in mature third instar neurons, likely in cooperation with actin." (p. 17)

(2) The authors characterize the localization of Shot during larval stages but not during pupal stages. The authors should characterize where Shot localizes during dendrite pruning, since it is possible that Shot may localize differently at this stage. For example, the authors mention that Shot localizes to dendrite tips during early larval stages but not late larval stages; it seems that the localization of Shot is dynamic.

Thank you for this comment. In response, we analyzed Shot localization in a time-course experiment including the second instar larval stage and at 5 h APF in the early pupal stage (new Figure S4). This showed that Shot localizes almost exclusively to dendrite tips at the first instar, but is more broadly distributed in the soma and along dendrites shafts at all later developmental stages.

New Fig. S4, time course of Shot::GFP localization including first to third instars and early pupal stage.

(3) Figure 3: Khc-lacZ is not an ideal read-out of microtubule polarity per se. Better, read-out microtubule polarity using EB1-GFP.

Thank you for this comment. Unfortunately, this experiment is not feasible in this situation. In our MARCM system, we use a red fluorescent tdTomato to label c4da neurons, and all Shot transgenes carry GFP tags, prohibiting the use of both fluorescent EB1 transgenes that we have, EB1::GFP and EB1::mScarlet3.

(4) Figure 4 A-A' (also Figure S4): Superresolution microscopy data are presented but not analyzed. Analysis should be included before drawing a conclusion from these data.

Thank you for this comment. In response, we quantified the width of the dendritic microtubule bundles in the STED experiments (now Fig. S5) and found that these bundles are significantly thinner upon shot knockdown.

New Fig. S5 B, quantification of STED data.

(5) Figure 5, A and B: These images are extremely difficult to interpret on their own. It is not clear what the authors are interpreting as a positive signal. Analysis and quantification of Shot-FL and Shot-deltaCH1 should be included, not just representative images. This seems like a missed opportunity.

Thank you for this comment. In response, we show higher magnification images of Shot(endo)GFP, UAS-Shot::GFP, and the two actin-binding mutants UAS-Shot ΔCH1::GFP and UAS-Shot ΔABD::GFP. For better interpretation, we indicate the boundaries of the dendrites as determined by tdTomato expression in the neurons (new Fig. 5 A - D).

(6) Figure 6: If gamma-tubulin is knocked down, does this decrease the number of EB1-GFP comets that originate from Rab11(+)-endosomes?

Thank you for this comment. This experiment is technically challenging because of the number and location of UAS transgenes involved. In addition, gamma-tubulin knockdown only has minor effects on pruning (no defects) or microtubule behavior (no orientation defects) in c4da neurons Wang et al., 2019, eLife (Fengwei Yu lab).

(7) Figure 6: Rab11(+) endosomes correlate with the initiation of EB1-GFP comets in first instar larvae, but the orientation of microtubules is analyzed in third instar larvae -is there an effect on microtubule polarity in younger larvae? What about second instar larvae? On a related note, what is the orientation of microtubules that originate from Rab11(+) endosomes? These should be quantified data.

Thank you for this comment. We analyzed the effect of Rab11 knockdown at the first instar stage and found that this led to a non-significant increase in anterograde comets. These data are now included as new Fig. S6. We also checked the orientation and found that all comets from Rab11-cherry puncta were retrograde, this is now mentioned in the text.

New Fig. S6, effect of Rab11 dsRNA on comet orientation in first instar neurons.

(8) Figure 6: Panel G shows that Rab11-GFP and Msps-FLAG co-immunoprecipitate. What effect does Rab11[S25N] have on EB1-GFP comet frequency and site of origin?

Thank you for this comment. Unfortunately, Rab11[S25N] does not cause c4da neuron dendrite pruning phenotypes in our hands, even though it has worked in another lab (Lin et al., 2020, PLoS Genetics), possibly due to subtle differences in the GAL4 transgenes used. We therefore did not try this transgene in our assays.

(9) Figure 6: Does Patronin colocalize with Rab11 in neurons?

Thank you for this comment. We had originally included an image of Patronin::GFP expressed in c4da neurons that appeared to show tip enrichment. When we tried to reproduce and quantify this result, we found that Patronin localization varied strongly with expression levels, and more focused or punctate signals seemed to reflect either aggregation or decoration of microtubules. We therefore removed the image and instead included a more detailed genetic analysis of Patronin, which shows similar phenotypes as Shot MT orientation defects at the first instar, enhancement of Shot knockdown phenotypes (Fig. 6).

Removed Patronin::GFP image (old Fig. 7H), added effect of Patronin dsRNA on comet orientation at first instar, enhancement of Shot dsRNA phenotype (new Fig. 6 D, E).

(10) Figure 7D: These data showing EB1-mScarlet3 comets originating from Shot-GFP should be quantified. Also, which Shot domains are involved in this recruitment?

Thank you for this comment. We observed only very few comets directly from tips in these experiments which precluded quantification. Acknowledging this, we moved the image to Supplementary Fig. 5.

As EB1 likely binds to Shot via SxIP motifs in the C-terminal region, we also tested whether the ShotΔCTail construct lacking the GAS2 domain and adjacent regions can recruit EB1. This is not the case. We therefore conclude that Shot recruits EB1 via its C-terminal part (new Figure 6D, E).

(11) Figure 2, A and B; Figure 4 B; Figure 6, B and D: Please include a scale bar for the time axis on these (and any other) kymographs.

Thank you for this comment. We checked and added time scale bars if missing.

(12) The authors should analyze the localization of GFP-tagged endogenous Shot in parallel to the exogenous expression of Shot under Gal4-UAS control.

Thank you for this comment. We tried to image endogenously tagged Shot::GFP at the first instar timepoint, but this was unfortunately not feasible due to very high Shot::GFP expression in neighboring tissues at this stage (and relatively low expression in c4da neurons).

(13) The authors should clarify at some point in the manuscript that EB1 comets can reflect either de novo microtubule growth (microtubule nucleation) or growth from the pre-existing ends of microtubules. EB1 comets alone do not indicate microtubule nucleation.

Thank you for this comment. We added the following sentence to the description of the first EB1 experiment (p. 7): "EB1 binds to the plus ends of growing microtubules (both newly nucleated and re-elongating), and plus end-bound EB1 is visible as moving dots (also known as comets)."

(14) It is unclear what the n in the graphs in the figures represent: neurons? Or dendrites/comets/etc? Please specify in the figure legend.

Thank you for this comment. We now specify this for each graph.

(15) Page 6, the authors write that "We and others have previously shown that c4da neuron dendrite pruning depends on local microtubule disassembly in proximal dendrites," but only cite their paper; please include the citations for the work by others.

Thank you for this comment. We added additional references.

(16) Page 9, the authors state: "Such phenomena are often seen when microtubules are not attached to specific anchoring sites such that they can be moved by microtubule motors." This is a fairly speculative interpretation of the data and should be saved for the Discussion.

Thank you for this comment. We moved this speculation to the Discussion.
